# PD221 Health Regulation And Health Technology Assessment: Brazilian Experience In Developing Actions For Health Regulation

**DOI:** 10.1017/S0266462324004409

**Published:** 2025-01-07

**Authors:** Flávia Tavares Silva Elias, Erika Barcosa Camargo, Margarete Martins de Oliveira, Maíra Catharina Ramos, Erica Tatiane da Silva

## Abstract

**Introduction:**

In 2017, the Brazilian Health Regulatory Agency (Anvisa) and the Oswaldo Cruz Foundation (Fiocruz) in Brazil entered into a collaboration that enabled the production of evidence and dissemination of technical scientific information in the regulatory field, with aim of supporting the decision-making process on regulatory issues.

**Methods:**

This qualitative and descriptive study was designed to report on the technical cooperation between Anvisa and Fiocruz in Brazil during from 2017 to 2020.

**Results:**

The studies developed by the Decentralized Execution Term assisted in the formulation of health policies at the macro, meso, and micro political levels, supporting the strengthening of institutional regulatory policy. The medicines agenda was the most recurrent in cooperation outputs, followed by the food and smoking agendas. The postmarket surveillance agenda played a leading role in actions carried out with the updating of the Technovigilance Manual and the creation of pharmacovigilance bulletins, both of which were available to health professionals and citizens. Finally, 27 courses were offered, and 1,276 certificates were issued (606 to workers linked to health regulation).

**Conclusions:**

The Brazilian experience enabled to capacity building and critical analysis of evidence in regulatory scope. It has facilitated the preparation of productions that align with the regulatory agenda. Health technology assessment and health regulation need to converge in monitoring processes to reduce uncertainties and increase user safety.

